# Exploring Changes to the Actionability of COVID-19 Dashboards Over the Course of 2020 in the Canadian Context: Descriptive Assessment and Expert Appraisal Study

**DOI:** 10.2196/30200

**Published:** 2021-08-06

**Authors:** Erica Barbazza, Damir Ivanković, Sophie Wang, Kendall Jamieson Gilmore, Mircha Poldrugovac, Claire Willmington, Nicolas Larrain, Véronique Bos, Sara Allin, Niek Klazinga, Dionne Kringos

**Affiliations:** 1 Department of Public and Occupational Health, Amsterdam UMC Amsterdam Public Health Research Institute University of Amsterdam Amsterdam Netherlands; 2 OptiMedis AG Hamburg Germany; 3 Hamburg Center for Health Economics University of Hamburg Hamburg Germany; 4 Laboratorio Management e Sanità, Institute of Management and Department EMbeDS Scuola Superiore Sant'Anna Pisa Italy; 5 Institute of Health Policy, Management and Evaluation University of Toronto Toronto, ON Canada

**Keywords:** COVID-19, performance measures, health information management, dashboards, public reporting of health care data, qualitative research, public health, medical informatics, surveillance, communication, assessment, Canada, decision-making, dynamic, development

## Abstract

**Background:**

Public web-based COVID-19 dashboards are in use worldwide to communicate pandemic-related information. Actionability of dashboards, as a predictor of their potential use for data-driven decision-making, was assessed in a global study during the early stages of the pandemic. It revealed a widespread lack of features needed to support actionability. In view of the inherently dynamic nature of dashboards and their unprecedented speed of creation, the evolution of dashboards and changes to their actionability merit exploration.

**Objective:**

We aimed to explore how COVID-19 dashboards evolved in the Canadian context during 2020 and whether the presence of actionability features changed over time.

**Methods:**

We conducted a descriptive assessment of a pan-Canadian sample of COVID-19 dashboards (N=26), followed by an appraisal of changes to their actionability by a panel of expert scorers (N=8). Scorers assessed the dashboards at two points in time, July and November 2020, using an assessment tool informed by communication theory and health care performance intelligence. Applying the nominal group technique, scorers were grouped in panels of three, and evaluated the presence of the seven defined features of highly actionable dashboards at each time point.

**Results:**

Improvements had been made to the dashboards over time. These predominantly involved data provision (specificity of geographic breakdowns, range of indicators reported, and explanations of data sources or calculations) and advancements enabled by the technologies employed (customization of time trends and interactive or visual chart elements). Further improvements in actionability were noted especially in features involving local-level data provision, time-trend reporting, and indicator management. No improvements were found in communicative elements (clarity of purpose and audience), while the use of storytelling techniques to narrate trends remained largely absent from the dashboards.

**Conclusions:**

Improvements to COVID-19 dashboards in the Canadian context during 2020 were seen mostly in data availability and dashboard technology. Further improving the actionability of dashboards for public reporting will require attention to both technical and organizational aspects of dashboard development. Such efforts would include better skill-mixing across disciplines, continued investment in data standards, and clearer mandates for their developers to ensure accountability and the development of purpose-driven dashboards.

## Introduction

The public reporting of data during a pandemic is a core government function to protect population health and safety [1-3]. It is also critical for fostering accountability, ensuring transparency, and supporting individuals in making informed decisions [4-6]. Unlike past pandemics, COVID-19 has been monitored globally in real-time, resulting in unprecedented collection, analysis, and dissemination efforts.

Public web-based COVID-19 dashboards, as a dynamic means to visually display information at a glance [7], have surged as a popular approach for sharing pandemic-related information. Dashboards are powerful vehicles for communication; the Johns Hopkins Coronavirus Resource Center dashboard [8] reported more than 1 billion interactions per day by April 2020 [9]. However, without careful indicator selection and data collection, analysis, and visualization, dashboards have the potential to mislead, misinform, and incite panic [10,11], or simply to be ignored [12].

In the first half of 2020, our international research network of European and Canadian professionals in health care performance intelligence [13] launched a global study of COVID-19 dashboards. It assessed 158 dashboards from 53 countries in July 2020. It also explored what makes dashboards *actionable*, whereby actionability refers to a dashboard’s potential to inform decision-making by the intended users [14]. More specifically, to be actionable, the information should be both *fit for purpose* (meeting a specific information need) and *fit for use* (placing the right information into the right hands at the right time and in a manner that can be understood) [14]. Only 12.7% (20/158) of dashboards evaluated in the mid-2020 study were found to be highly actionable. Seven actionability features were identified among them [15].

Due to the speed at which the dashboards were first launched, traditional technical and organizational aspects of development cycles were cut short [16]. While the urgency of reporting took precedent in the early stages, dashboards are designed to be flexible and continuously iterated. Studies also emphasized the importance of frequent reviews to ensure a dashboard’s sustained relevance and use [16,17]. As our initial study was merely a snapshot of the early stages of the pandemic, the extent to which COVID-19 dashboards evolved over a longer period was beyond its scope.

Canada provides a relevant context for further investigating the evolution of COVID-19 dashboards for several reasons. First, public health is the remit of federal, provincial or territorial (PT), and local health authorities [18], which, together with PT ministries, are involved in pandemic monitoring and reporting. This was already reflected in Canada’s 2018 multiactor pandemic preparedness plans (for influenza) [19]. In addition to those varied public actors, independent initiatives and the media have also leveraged open data sources in order to generate public-facing COVID-19 dashboards. The range in the types of organizations and their different target geographies of reporting have resulted in a diverse Canadian dashboard landscape.

Second, Canada’s experience with COVID-19 intensified in the course of 2020, with an initial peak in early May (about 2500 daily cases) and second peak in November (about 8000 daily cases) [20]. Cases spread to areas of Canada previously untouched by the virus [21]. As a result, the demand for dashboards that provide effective communication and support data-driven decision-making increased throughout the year.

Third, Canadian dashboards were criticized early on for possible information blind spots, including a failure to report race-based data and other social determinants [22,23], as well as for presenting highly aggregated data at the PT level [10,24,25]. The extent to which such limitations persisted into the second half of 2020 is yet to be assessed.

This study explores (1) how public web-based COVID-19 dashboards in the Canadian context evolved in 2020 and (2) whether dashboard actionability increased over time.

## Methods

### Study Design

Our study adheres to the Standards for Reporting Qualitative Research [26]. We applied qualitative methods comprising (1) a descriptive assessment applying an existing tool [15] for the purposes of systematically and comparatively depicting COVID-19 dashboards; and (2) an expert appraisal using the nominal group technique [27,28] to score the actionability of the dashboards. The study draws on the global sample of 158 dashboards examined in the study by Ivanković et al [15], now confining the focus to dashboards reporting on COVID-19 in the Canadian context (N=26). Importantly, we extended data collection for this sample by collecting data at a second time point, in order to analyze changes between July 2020 (initial assessment) and November 2020 (second assessment). Subsequently, we evaluated the presence of the actionability features identified in the study by Ivanković et al [15] across the sample for both time points.

### Panel of Scorers

Data collection was conducted by a panel of eight scorers (EB, DI, SW, KJG, MP, CW, NL, and VB). The panel (four women and four men) aligned with the scorers assembled by Ivanković et al [15] so as to ensure consistency between assessments. The scorers were drawn from an existing international research network of Canadian, European, Latin American, and Asian researchers, each conducting their doctoral research on health care performance intelligence [13]. All scorers had common expertise and training in dealing with health care performance data and in the use of such data for management and governance, as well as prior training and experience with the study’s assessment tool. The panel’s composition also included French-language competencies (CW) and prior professional policy and research experience in the Canadian context (EB, DI, SW, KJG, MP, and VB).

### Assessment Instruments

An assessment tool developed, piloted, and validated by Ivanković et al [15] was applied. The tool assesses COVID-19 dashboards in terms of their purpose and users (“why”), content and data (“what”), and analyses and displays (“how”). Table 1 summarizes the considerations assessed. These derive from communication sciences (the 1948 Lasswell model [29]), the health care performance intelligence discipline [14], earlier studies on the public reporting of health performance data and provision of dashboards in the health domain [30-34], and guidance for reporting during public health crises from the World Health Organization (WHO) [1]. The tool also aligns with existing instruments to measure the quality of health information on the internet [35,36].

We operationalized the appraisal of a dashboard’s actionability by drawing on the seven features of highly actionable COVID-19 dashboards, as identified in the study by Ivanković et al [15] (see Table 1). A scoring tool was developed (see Multimedia Appendix 1) to evaluate each feature on a 3-point ordinal scale, scored as “present,” “somewhat present,” or “not present.”

**Table 1:** Overview of considerations by the method applied.

Method	Instrument	Considerations assessed/scored: guiding questions/statements
Descriptive assessment	Assessment tool^a^	Purpose and audience: Is the purpose and audience mentioned? Indicator themes: What indicators are reported on? Data: Are data sources and metadata specified? Types of analysis: Does the analysis include time trends, and geographic and population break downs? Presentation: How is data visualized, interpreted, simplified, and interacted with?
Expert appraisal	Seven features of highly actionable dashboards-scoring tool^b^	Know the audience and their information needs: The intended audience and their information needs are known and responded to. Manage the type, volume, and flow of information: The type, volume, and flow of information on the dashboard are well managed. Report data sources and methods clearly: The data sources and methods for calculating values are made clear. Link time trends to policy decisions: Information is reported over time and contextualized with policy decisions made. Provide data “close to home”: Data are reported at relevant geographic break downs. Break down the population to relevant subgroups: Data are reported by relevant population subgroups. Use storytelling and visual cues: Brief narratives and visual cues are used to explain the meaning of data.

^a^Refer to the study by Ivanković et al [15] for the full assessment tool.

^b^Refer to Multimedia Appendix 1 for the full scoring tool.

### Study Sample

COVID-19 dashboards for sample inclusion were determined on the basis of the following three criteria: (1) the reporting of key performance indicators related to COVID-19; (2) the use of some form of visualization; and (3) availability in an online web-based format. It means password-protected COVID-19 dashboards for internal use by public authorities were excluded from this study. No restrictions were imposed in terms of a dashboard’s primary level of reporting (eg, national, regional, and local) or the type of organization responsible for its development (eg, government, academia, news or media, industry, and private initiative). Sampling was conducted from May 19 to June 30, 2020, and involved searches of COVID-19 policy monitoring platforms (eg, the North American COVID-19 Policy Response Monitor [37]) and of research reports (eg, a June 2020 pan-Canadian catalogue of governmental COVID-19 dashboards [38]), as well as expert recommendations from researchers actively engaged in the COVID-19 response, who were contacted via email. In total, 31 dashboards reporting on the Canadian context were identified, five of which were duplicates and excluded from further analysis. Further details about the sampling are mentioned in the study by Ivanković et al [15].

**Figure 1:**
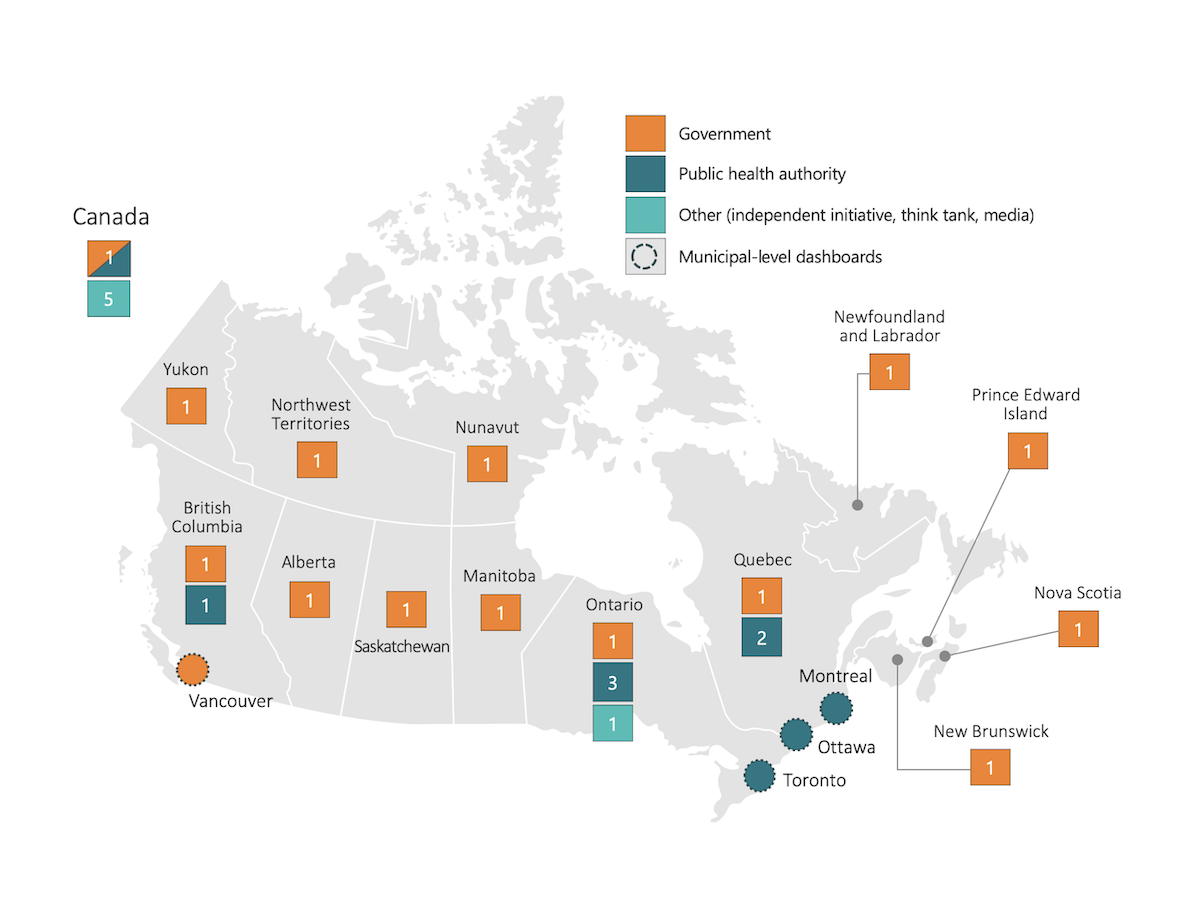
Distribution of COVID-19 dashboards sampled and types of organizations responsible for their development. Circles denote municipal-level dashboards included in the sample, and the colors denote the respective organization types. These dashboards are counted in the tally shown per jurisdiction. The Public Health Agency of Canada’s COVID-19 dashboard is hosted on the federal Government of Canada webpage. In other instances, dashboards developed by public health authorities are hosted on dedicated webpages.

The final sample (N=26) included dashboards reporting at the national level (n=6), PT level (n=16) (including at least one from each of Canada’s 13 provinces and territories), and municipal level (n=4), capturing reporting from the capital (Ottawa) and the three largest cities (Montreal, Toronto, and Vancouver). Figure 1 maps the pan-Canadian distribution and the variations in the types of organizations responsible for developing the dashboards. These included federal or PT governments (14/26, 54%), public health authorities (6/26, 23%), and others (6/26, 23%), including independent initiatives (eg, #HowsMyFlattening and COVID-19 Canada Open Data Working Group), industry (eg, Esri and Deloitte), and media (Canadian Broadcasting Corporation). See Multimedia Appendix 2 for the complete list of dashboards.

### Descriptive Assessment

Each dashboard was assessed in English or French. The assessments were limited to a dashboard’s main page and to content accessible within one interaction (click). This approach was designed to increase consistency in the content evaluated, and it enabled us to gauge the dashboard’s prioritization and hierarchy of content. Archives were generated to create a record of each dashboard on the date reviewed (see Multimedia Appendix 2). Dashboards were distributed among the scorers as described in the study by Ivanković et al [15]. This distribution (averaging three dashboards per scorer) remained consistent between time points as follows: the same scorers assessed the same dashboards in both July and November 2020. All assessments additionally underwent reviews by the first authors (EB and DI) to verify completeness and consistency.

### Expert Appraisal

To assess the presence of the seven defined features of highly actionable COVID-19 dashboards, we organized a series of three-person panels, involving the original scorer of each dashboard joined by two other experts (the first authors or another panel member), in December 2020. Prior to the start of the appraisal by each panel, a workshop with the scorers was organized to calibrate the approach to scoring.

Scoring was informed by the original data records and archives generated in the two descriptive assessments (July and November 2020). Importantly, each of the seven actionability features were appraised with consideration to the dashboard’s stated or inferred purpose and audience. It means the appraisal of each feature differentiated between the intended use of the dashboard by national, PT, or municipal general public audiences, unless further specified. In line with the nominal group technique approach [27,28], the three panel members first independently scored the presence of each feature on the dashboard using the scoring tool described above. The proportion of identical ratings for each dashboard was calculated, and virtual panel discussions were convened between the three scorers involved [39,40].

Prior to those discussions, partial or full agreement (two- or three-way consensus) had been reached on 83.5% (304/364) of the items scored, with full three-way agreement on 50.0% (182/364) (see Multimedia Appendix 3). During the panel discussions, all items without full agreement were debated. All panels reached final agreement by discussion or re-examining the data records or archives.

### Data Analysis

We used descriptive statistics to analyze the data at the two time points. We first determined the number and percentage of dashboards in which each item (ie, each consideration) of the descriptive assessment had been recorded as present in the July or November assessment or both. The net change for each item was calculated as the change in the total number of dashboards and the direction of that change between time points. To analyze score changes for the actionability features, we calculated feature-by-feature totals in both July and November, applying a 3-point ordinal scale (not present, somewhat present, and present). Using the same approach applied to analyze changes over time in the descriptive assessments, we calculated the net change per feature as the change in the total number of positively scored dashboards, noting the direction of that change.

For free-text fields in the descriptive assessment tool, we used both deductive and inductive thematic analysis to identify themes [41,42]. This applied to responses on considerations such as a dashboard’s purpose of use and audience, indicator titles, and considerations with “other” as an answer category. Topics explored in the assessment tool were used to guide the deductive thematic analysis. In analyzing the titles of indicators reported by the dashboards, we applied the existing WHO classification of types of pandemic-related information. Indicators were analyzed by the types of information as follows: public health and epidemiology, health system management, social and economic impact, and behavioral insights [1]. Given the observed variability in the phrasing of indicator titles, the first authors grouped key performance indicators by themes. New themes that emerged were identified using an inductive approach.

### Ethics Approval

This study involved the analysis of publicly available COVID-19 dashboards. Ethics approval was not required.

## Results

### Sampled Dashboards

The 26 Canadian COVID-19 dashboards were assessed in the time frames July 7 to July 20 and November 23 to December 2, 2020, with an average of 135 days between assessments (range 132-140). All dashboards remained active, with regular, typically daily updating, aside from one (City of Vancouver), which was still accessible but last updated in August 2020. As expected, given the wide differences in population size and density across Canadian provinces and territories, the cumulative number of COVID-19 cases reported by the dashboards for their respective geographic areas ranged from 0 cases in Nunavut to more than 55,000 in Quebec in July, and from 15 cases in Northwest Territories to more than 140,000 in Quebec in November. Cumulative numbers of COVID-19 cases and deaths on the assessment dates are reported in Multimedia Appendix 2.

### Changes to the Dashboards Over Time

Table 2 reports how the dashboards changed over time according to the descriptive assessment. The changes can be summarized as presented below.

**Table 2:** Description of changes to Canadian COVID-19 dashboards (N=26) over time in 2020.

Consideration and description					July value, n (%)			November value, n (%)			Net change^a^
**Purpose and audience**											
	Purpose: Purpose of use of the dashboard stated			10 (39%)			10 (39%)			0	
	Audience: Intended audience (user) stated			3 (12%)			4 (15%)			+1	
**Indicator themes**											
	**Spread and death**										
		Cases (all confirmed cases)	25 (96%)			25 (96%)			0		
		Deaths	20 (77%)			21 (81%)			+1		
		Recovered (healed, cured)	17 (65%)			18 (69%)			+1		
		Active cases	12 (46%)			12 (46%)			0		
		Mortality rate (case fatality rate)	4 (15%)			4 (15%)			0		
		Reproduction rate (attack rate)	1 (4%)			5 (19%)			+4		
	**Testing**										
		Testing (total number tested, PCR^b^ tests)	17 (65%)			19 (73%)			+2		
		Testing rates (positivity, negative tests)	10 (39%)			15 (58%)			+5		
		Tests pending results	4 (15%)			2 (8%)			−2		
		Testing turnaround	0 (0%)			3 (12%)			+3		
	**Risk management**										
		Self-quarantine (isolation notices)	1 (4%)			1 (4%)			0		
		Contact tracing	2 (8%)			2 (8%)			0		
	**Hospital care**										
		Hospitalized (admissions, discharges)	16 (62%)			15 (58%)			−1		
		Admitted to the ICU^c^ (critical condition)	10 (39%)			12 (46%)			+2		
		On a ventilator	3 (12%)			3 (12%)			0		
	**Health system capacity**										
		Hospital bed capacity (availability)	2 (8%)			2 (8%)			0		
		ICU bed capacity	3 (12%)			2 (8%)			−1		
		Ventilator capacity (available ventilators)	3 (12%)			2 (8%)			−1		
		Non-COVID-19 service usage	1 (4%)			1 (4%)			0		
		Personal protective equipment stock	1 (4%)			1 (4%)			0		
	**Economic/social impact**										
		Employment and hardship relief	4 (15%)			4 (15%)			0		
		Transport, trade, and international travel	2 (8%)			3 (12%)			+1		
		Behavioral: Public risk perception/restriction adherence	5 (19%)			3 (12%)			−2		
	**Other**										
		Future projections (modelling)	1 (4%)			1 (4%)			0		
		Risk-level/current phase (composite score)	2 (8%)			4 (15%)			+2		
**Data sources and metadata**											
	Sources: Data sources are noted			18 (69%)			18 (69%)			0	
	Metadata: Metadata are specified			11 (42%)			14 (54%)			+3	
**Types of analyses**											
	**Time trend**										
		Time trend analysis available	21 (81%)			23 (89%)			+2		
		Customizable time trend	4 (15%)			10 (39%)			+6		
	**Number of geographic levels**										
		1 level	6 (23%)			3 (12%)			−3		
		2 levels	14 (54%)			15 (58%)			+1		
		3 or more levels	6 (23%)			8 (31%)			+2		
	**Types of geographic levels of analysis**										
		International	3 (12%)			3 (12%)			0		
		National	9 (35%)			8 (31%)			−1		
		Regional (province/territory)	22 (85%)			22 (85%)			0		
		Health regions	10 (39%)			15 (58%)			+5		
		Municipal (city)	8 (31%)			8 (31%)			0		
		Neighborhood (postcode)	3 (12%)			2 (8%)			−1		
	**Disaggregation options**										
		Age	18 (69%)			17 (65%)			−1		
		Sex	14 (54%)			15 (58%)			+1		
		Mode of transmission	5 (19%)			6 (23%)			+1		
		Long-term care facilities	5 (19%)			5 (19%)			0		
		Schools	2 (8%)			5 (19%)			+3		
		Ethnicity	0 (0%)			2 (8%)			+2		
		Race	0 (0%)			2 (8%)			+2		
		Comorbidities	1 (4%)			1 (4%)			0		
		Socioeconomic status	1 (4%)			1 (4%)			0		
		Health workers	3 (12%)			1 (4%)			−2		
**Presentation**											
	**Type of visualization**										
		Table	20 (77%)			25 (96%)			+5		
		Graph/chart	21 (81%)			22 (85%)			+1		
		Map	15 (58%)			18 (69%)			+3		
	**Narratives to interpret data**										
		Yes, to clarify the quality of the data	13 (50%)			18 (69%)			+5		
		Yes, to clarify the meaning of the data	12 (46%)			11 (42%)			−1		
	**Simplification techniques**										
		Use of color coding	15 (58%)			15 (58%)			0		
		Size variation	3 (12%)			4 (15%)			+4		
		Icons	3 (12%)			7 (27%)			−2		
	**Interactive options**										
		More information	18 (69%)			18 (69%)			0		
		Change of information	7 (27%)			10 (39%)			+3		
		Change of display	5 (19%)			6 (23%)			+1		

^a^Net change refers to the total number of dashboards and the direction of overall change between time points. Importantly, no net change (0) can mean both no change or the same number of dashboards increased and decreased for the specific consideration.

^b^PCR: polymerase chain reaction.

^c^ICU: intensive care unit.

### Purpose and Audience

There was no change in the extent to which dashboards stated their purpose of reporting, with just over one-third (10/26, 38%) doing so in both July and November. Where stated, the most frequent specific aims of dashboards were to provide simplified information in an “easy-to-digest, actionable way” [43] and to “help prevention strategies reach those people most affected” [44]. The explicit mention of a target audience was even less frequent, being found on just four dashboards (4/26, 15%) in November, a marginal increase from July (3/26, 12%). Target audiences were denoted as “general public,” “businesses,” or “public health leaders.” Notable improvements over time were made by Ontario’s #HowsMyFlattening [43], with the introduction of two dashboard viewing modes (“personal” and “geek”) to serve the information needs of different audiences.

### Indicator Themes

Across the dashboards, public health and epidemiological indicators, followed by health system management indicators, were the most frequently reported indicators at both time points. Behavioral and socioeconomic indicators were rare. An average of seven indicator themes were reported per dashboard in November (range 2-17), compared with six in July (range 2-15). Several indicators became more prevalent in November, including viral reproduction rates, testing rates, testing turnaround times, and composite scores. Six dashboards (6/26, 23%) reduced the number of indicator themes reported, most often removing indicators on active cases. In some instances, indicators had been moved from the dashboard to new tabs or pages, as in Ottawa [45], which relocated indicators on behavioral insights to new tabs no longer within direct access of the main dashboard page assessed. Indicators on serology tests, doubling rates, and testing stock, which had been present on dashboards previously assessed internationally [15], were not reported at either time point on the sampled dashboards.

### Data Sources and Metadata

A third (8/26, 31%) of the dashboards, all government developed, did not explicitly report data sources in July or November. Dashboards typically drew data from jurisdiction-specific health services and public health authorities, hospital databases, and, for comparisons with other countries, the Johns Hopkins University Coronavirus Resource Center dashboard. Dashboards reporting metadata (supplementary details on the calculation of the indicators) increased to more than 50% (14/26, 54%) by November (from 11/26, 42%, in July). Notably, the COVID-19 in Canada dashboard published a detailed technical report on its data set produced by the COVID-19 Canada Open Data Working Group initiative [46,47].

### Types of Analyses

A slight increase in the number of dashboards reporting time-trend data was observed between July and November (from 21/26, 81% to 23/26, 88%). Improvements were also made to the availability of customizable time scales, allowing users to zoom in on specific time frames of interest (from 4/26, 15% to 10/26, 38%).

Modifications were made to report subregional geographic breakdowns of data, with more than half (15/26, 58%) of the dashboards including breakdowns by health regions in November, as compared with 10 (10/26, 38%) in July. Age and sex remained the most common population breakdowns in November (17/26, 65%, as against 15/26, 58%, in July), followed by mode of transmission (6/26, 23%) and long-term care facilities (5/26, 19%). Schools emerged as a new type of breakdown in November, though present on only one-fifth (5/26, 19%) of dashboards.

### Presentation

Between July and November, most dashboards slightly improved the number and variety of chart types, simplification techniques, and interactive features they made available. This was mostly done by introducing maps or additional tables and icons, as well as user-directed modifications to the information displayed. New features that emerged in November included options to subscribe to email updates for alerts (eg, #HowsMyFlattening [43] and Ottawa [45]). Two dashboards (Quebec [48] and Ontario [49]) introduced user feedback surveys.

Text providing details on data quality was present on more than two-thirds (18/26, 69%) of dashboards in November, compared with half (13/26, 50%) in July. For example, Esri’s dashboard included lay-language explanations of values with statements such as “*Why do I sometimes see negative numbers?* Some values reported (like total cases) are cumulative. They always go up. Other values (like hospitalizations) fluctuate and can go up or down day-to-day” [50]. Narratives to explain the meaning of statistics and trends were provided by fewer than half (11/26, 42%) of the dashboards in November. Explanations of trends and their meaning included the following description provided by the COVID-19 in Canada dashboard: “Graphs display trends for daily cases and deaths over time on a logarithmic scale. An upward slope means the number of cases/deaths reported each day is still growing. A flat line means the number of cases/deaths reported each day is staying the same. A downward slope means the number of cases/deaths reported each day is falling” [20].

### Actionability Features Over Time

Of the 26 dashboards assessed, none was found to fully present all seven of the defined actionability features either in July or November. Overall, 8% (2/26) of dashboards were assessed in July as having five or more actionability features fully present, doubling to 15% (4/26) of dashboards in November. Three quarters (77%, 20/26) of dashboards had two or fewer features fully present in July and 65% (17/26) had two or fewer features fully present in November. Seven dashboards increased their score of fully present features. Although two dashboards scored lower in November, the decrease was largely attributable to modifications in the type of information reported on the main dashboard page, as indicators were moved to other dedicated pages.

**Figure 2:**
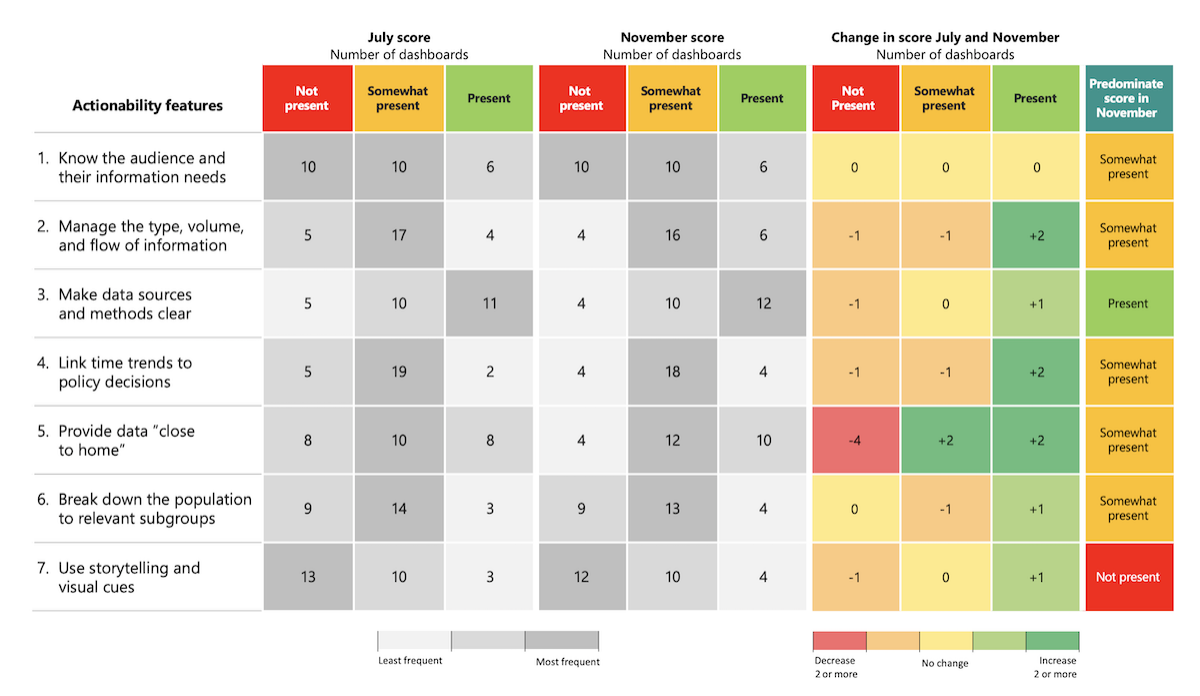
Change in actionability across dashboards (n=26) over time in 2020. Not present: the feature is not found on the dashboard; somewhat present: some elements of the feature are present on the dashboard but room for improvement; present: the specific feature is clearly demonstrated and a good practice example of the feature is present. See Multimedia Appendix 1 for full scoring details and Multimedia Appendix 3 for the level of agreement between panel members.

The actionability feature most widely present on dashboards in both July and November was the clarity of data sources and methods, while the use of storytelling and visual cues was the feature most frequently absent (Figure 2)*.* Among the seven defined features of actionability, improvements were observed in all but one (knowing the audience and their information needs), which was present on fewer than a quarter of the dashboards at either time point. Improvements were most pronounced for the feature involving geographic breakdown, with average scores increasing by nearly a quarter from July to November. Second to these improvements were improvements in the use of time trends, although explicit links between data and policy decisions and infection control measures remained infrequent.

## Discussion

### Principal Findings

In this study, we explored changes made in the course of 2020 to public web-based COVID-19 dashboards in Canada and appraised their actionability for decision-making purposes. Although the dashboards we sampled varied in their specific geographic focuses, they all shared an increasing relevance in supporting data-driven decision-making in their respective audiences as the severity of the COVID-19 pandemic intensified across the country. Broadly speaking, from the perspective of the health care performance intelligence we applied, we observed that subtle improvements were made to the dashboards between July and November 2020. Improvements were most pronounced with regard to dashboard technology solutions (better customizable time trends, and new charts and graphs) and data provision (new indicators, more transparency on metadata, and more geographic granularity). Modifications to further develop communicative elements were less pronounced or even absent during the period assessed. These results were mirrored in the scoring of actionability features.

COVID-19 dashboards worldwide are powered by a somewhat common range of software service providers (eg, ArcGIS, Tableau, and Power BI). We presume that some improvements observed across our sample can be credited to new technical features rolled out by such providers during 2020. For example, the use of adjustable time trends was a feature introduced on more than a third of the dashboards by November and was evidently an added element in the underlying software. However, while the industry may be credited with spearheading the technical development of dashboards, the current practice from a technological perspective of measuring actionability through *user clicks* [51] exposes some limitations. To give an example, the enhanced sophistication of the technology behind more interactive time trends used on dashboards was not complemented with improvements to incorporate the enactment of policy restrictions into time-trend graphs so as to visualize subsequent effects of those restrictions. This was despite the merits of such a visualization [15] and the fact that such a technique was already being applied on dashboards in countries like Australia [52] and Slovenia [53]. In our sample, we did observe dashboards that excelled in actionability, successfully leveraging the skills of specialists in technology, data, public health, and communication [43,54]. This finding is consistent with the findings in previous studies that have shown the importance of diverse stakeholder engagement for achieving actionable performance measurement, data reporting, and dashboard use [55-57]. In future research, we intend to further explore the perspective of dashboard developers, including their team profiles.

Improved geographic granularity and transparency of methods may be supported by initiatives like the COVID-19 Canada Open Data Working Group [20]. The overall subtlety of changes in available data and its specificity might be a symptom of underlying system barriers, in particular in relation to the collection and reporting of disaggregated data [58]. Researchers in the Canadian context have called attention to data management issues arising from unharmonized privacy laws, public/private data custodianship, and obstacles to the reuse of data for research [59]. The collection of race-based data in Canada is fragmented [60], and a pan-Canadian standard was proposed only in July 2020 [61]. There is a responsibility to act in cases where missing data could be masking inequitable burdens of the pandemic [62,63]. The potential equity-promoting impact of subpopulation-based approaches to the analysis and use of data has already been highlighted in Toronto [64]. Countries that report race- and ethnicity-based COVID-19 data, like New Zealand [65] and the United States [66], may be a source of insights into necessary data governance standards, privacy protections, and data infrastructure.

Our findings also reveal a responsiveness to the evolving nature of the pandemic, with multiple dashboards adding school cases or outbreaks as a data disaggregation option and turnaround times for virus testing as an indicator. Shortly after our second assessment, many dashboards also began reporting on vaccinations. Less advanced dashboards, from areas not seriously affected by the pandemic in the spring of 2020, made considerable progress in the second half of the year, as COVID-19 became more widespread. While such changes confirm that dashboards continued developing with time, the clarity of their intended aims and audiences nevertheless remained an underdeveloped attribute, despite wide recognition of the fundamental importance of data driven by a clear purpose and information need [14,67-70]. This may be a symptom of data governance constraints or, more specifically, of unclear responsibilities and mandates delegated to developers, as evidenced by the multiple public actors (eg, PT governments and PT public health authorities) that were reporting on the same geographies with nearly equivalent content. Although COVID-19 dashboards began as a need-based short-term tool for monitoring and communicating on the pandemic, this function has evolved with time. Dashboards must now face the mid-term challenge of dual-track health system monitoring, reporting both on the pandemic and on non-COVID health care [71], as well as the long-term challenge of integration into standard health system performance measurement. Rethinking the development of dashboards governed by clear mandates will be essential to ensure that relevant high-quality information is transparently delivered to well-defined audiences.

### Strengths and Limitations

To our knowledge, this is the first study to comparatively explore and critically reflect on changes to COVID-19 dashboards over time from a health care performance intelligence perspective. The study was enriched by the expertise of the panel, whose members had prior experience in assessing COVID-19 dashboards internationally, as well as a shared reflexive lens to gauge both the technical and communication aspects of the dashboards. Additionally, given the sustained relevance of COVID-19 dashboards, our findings are pertinent to both short-term improvements in COVID-19 dashboards and their longer-term utility in addressing future public health crises.

We acknowledge several limitations. First, the stages of the pandemic and its severity varied considerably across our sample, possibly contributing to differences with respect to the data available and the prioritization of a dashboard’s development. Despite this, the general direction of change was found to be common, averaging a three-fold increase in COVID-19 cases across locations between our assessment time points (see Multimedia Appendix 2). Second, the expert-based appraisal of actionability we employed is not a guaranteed reflection of a dashboard’s use in practice. The first-hand experiences of dashboard users merit further study to obtain practical real-world insights that can complement the concepts explored here. Third, our archiving of dashboards was limited to their main page. Dashboards with multiple tabs could therefore not be revisited in full for scoring purposes. To minimize the potential loss of information, all dashboards were assessed and evaluated by the same scorer in both July and November. Lastly, to permit comparisons over time, our sample was limited to dashboards identified in our search in May 2020. Any new dashboards that followed would have been missed. An exhaustive sample was beyond the study’s aims; however, we achieved geographic representativeness, as well as reasonable diversity in level (national, jurisdictional, and municipal) and in the types of providing organizations.

### Conclusion

Actionable dashboards are needed to enable effective decision-making across audiences. Dashboards are tools of continuing importance during the COVID-19 pandemic, but sustaining their actionability requires responsiveness to the pandemic’s stages. Improvements made to COVID-19 dashboards in the Canadian context from July to November 2020 appear to be driven mainly by certain technological and data improvements. The effective use of communication features remained underdeveloped at both points in time. COVID-19 dashboard developers need to better leverage the expertise of public health and communication specialists, in order to ensure that data will truly become information that is readily accessible and relevant to a public audience. Strategic system improvements to prioritize data standards, for example, those with respect to subpopulation-based data, are needed to achieve more significant gains in actionability. As the pandemic continues to evolve, attention will need to shift toward converting dashboards from their initial status as temporary monitoring and communication tools into instruments that are integrated into routine health system performance monitoring. Accomplishing that will also require improved governance arrangements that clarify roles and responsibilities. In the short term, continued improvements are urgently needed with respect to all seven of the identified actionability features, in order to make COVID-19 dashboards more fit for their purpose and use.

## Appendix

Multimedia Appendix 1 Scoring tool on actionability features.

Multimedia Appendix 2 Overview of Canadian COVID-19 dashboards assessed.

Multimedia Appendix 3 Scoring distribution and extent of agreement prior to joint workshops.

## Abbreviations

PT: provincial/territorial
WHO: World Health Organization
