# Supplementary material for: Exploring Changes to the Actionability of COVID-19 Dashboards Over the Course of 2020 in the Canadian Context: Descriptive Assessment and Expert Appraisal Study
Source: J Med Internet Res. 2021 Aug 6;23(8):e30200. doi: 10.2196/30200 (PMC8360335; doi:10.2196/30200)
Supplement: Multimedia Appendix 1 [file jmir_v23i8e30200_app1.docx]

**Multimedia Appendix 1**

Scoring tool on actionability features

| **Feature**^a^ | **Question/statement** | **Answer options** | **Description** | **Rationale** |
| --- | --- | --- | --- | --- |
| 1. Know the audience and their information needs | The intended audience and their information needs are known and responded to.  *Select one* | Present  Somewhat present  Not present | Considerations to look for: clear guiding key questions or aims; use of overall composite scores; clear intended audience; user-facing conveniences like multi-language functionality and exact timing of updating. | Dashboards with a known audience and explicit aim have better focus and continuity in their content, analysis and delivery. |
| 2. Manage the type, volume, and flow of information | The type, volume and flow of information on the dashboard is well managed.  *Select one* | Present  Somewhat present  Not present | Considerations to look for: manageable number of indicators; logical flow of information (general to specific, clustered as themes, etc.; indicators spanning types of information (epidemiological, health systems, social/economic, behavioural insights). | The selection of a concise number of indicators, yet ranging types of information, brings focus and importance to the information and the possibility to view indicators together, at-a-glance. |
| 3. Make data sources and methods clear | The data sources and methods for calculating values are made clear.  *Select one* | Present  Somewhat present  Not present | Considerations to look for: clear reporting of data sources; available (on page or redirected to) metadata data, data dictionary or similar; brief explanations (short narratives) that explain to a user about the data. | A clear source of data and explanation of an indicator’s construction, including potential limitations, is an important component of trust in the dashboard and clarity in its reporting. |
| 4. Link time trends to policy decisions | Information is reported over time and contextualized with policy decisions.  *Select one* | Present  Somewhat present  Not present | Considerations to look for: data is reported over time; time trends are customizable and can be tailored to a specific period of interest; policy decisions and their implications on rates is communicated. | Reporting data over time together with the introduction of key infection control measures facilitated an understanding of their effect (or lack of). |
| 5. Provide data ‘close to home’ | Data is reported at relevant geographic breakdowns.  *Select one* | Present  Somewhat present  Not present | Considerations to look for: information is broken down to levels relevant for the dashboard (e.g. city dashboard includes reporting at the municipal-level but also by boroughs and/or zip codes). | To inform individuals of risks in their immediate surroundings, granular geographic breakdowns are needed. Data that is highly aggregated is difficult to understand. |
| 6. Break down the population to relevant sub-groups | Data is reported by relevant population sub-groups.  *Select one* | Present  Somewhat present  Not present | Considerations to look for: data is reported by a range of breakdowns (e.g. sex, age, ethnicity, co-morbidities, etc.); breakdowns can be analyzed in ways that signal the relative risk of different population sub-groups. | Providing data with the possibility to explore varied population characteristics makes indicators relatable to individual users. It allows an understanding of risks and trends based on one’s own demographics. |
| 7. Use story-telling and visual cues | Brief narratives and visual cues are used to explain the meaning of data.  *Select one* | Present  Somewhat present  Not present | Considerations to look for: brief explanations are provided on the meaning of trends; use of visual techniques, such as intuitive color schemes and icons; effective display of information for a visually pleasing dashboard that facilitates an understanding of key figures. | A concise narrative explaining the significance of a trend supports users to understand the importance of the information. Bare statistics without a narrated analysis leave the burden of interpretation solely to the user. |

^a^Features are derived from the findings reported in: Ivanković D, Barbazza E, Bos V, Brito Fernandes Ó, Jamieson Gilmore K, Jansen T, Kara P, Larrain N, Lu S, Meza-Torres B, Mulyanto J, Poldrugovac M, Rotar A, Wang S, Willmington C, Yang Y, Yelgezekova Z, Allin S, Klazinga N, Kringos D. Features Constituting Actionable COVID-19 Dashboards: Descriptive Assessment and Expert Appraisal of 158 Public Web-Based COVID-19 Dashboards. J Med Internet Res 2021;23(2):e25682 doi: 10.2196/25682.
