# Supplementary material for: Exploring Changes to the Actionability of COVID-19 Dashboards Over the Course of 2020 in the Canadian Context: Descriptive Assessment and Expert Appraisal Study
Source: J Med Internet Res. 2021 Aug 6;23(8):e30200. doi: 10.2196/30200 (PMC8360335; doi:10.2196/30200)
Supplement: Multimedia Appendix 2 [file jmir_v23i8e30200_app2.docx]

**Multimedia Appendix 2**

Overview of Canadian COVID-19 dashboards assessed

| **#** | **Location**  Link | **Level** | **Type** | **Organization** | **Audience** | **Review dates** | **Archive by review date** | **Cases on review date** | **Deaths on**  **review date** |
| --- | --- | --- | --- | --- | --- | --- | --- | --- | --- |
| 1 | [Canada](https://health-infobase.canada.ca/covid-19/epidemiological-summary-covid-19-cases.html#a5) | National | Government/ public health authority | Government of Canada | General public^b^ | 13.07.2020 | <http://archive.vn/DW6Ow> | 107,590 | 8,783 |
|  |  |  |  |  |  | 23.11.2020 | <https://archive.vn/MrS33> | 330,503 | 11,455 |
| 2 | [Canada](https://resources-covid19canada.hub.arcgis.com/) | National | Other | Esri Canada | General public, data specialists^b^ | 13.07.2020 | <http://archive.vn/ujVQi> | 107,589 | 8,783 |
|  |  |  |  |  |  | 23.11.2020 | <https://archive.vn/sl4bp> | 333,803 | 11,494 |
| 3 | [Canada](https://art-bd.shinyapps.io/covid19canada/) | National | Other | Jean-Raul R Soucy and Isha Berry (Independent) | General public, data specialists^b^ | 13.07.2020 | <http://archive.vn/m2dLJ> | 109,920 | 8,827 |
|  |  |  |  |  |  | 23.11.2020 | <https://archive.vn/2E9md> | 333,560 | 11,483 |
| 4 | [Canada](https://newsinteractives.cbc.ca/coronavirustracker/) | National | Other | Canadian Broadcasting Corporation | General public^b^ | 13.07.2020 | <http://archive.vn/p7Ix2> | 108,278 | 8,825 |
|  |  |  |  |  |  | 23.11.2020 | <https://archive.vn/esdVA> | 330,503 | 11,454 |
| 5 | [Canada](https://covid19tracker.ca/) | National | Other | Noah Little (Independent) | General public^b^ | 18.07.2020 | <http://archive.vn/Lqk6L> | 99,537 | 8,104 |
|  |  |  |  |  |  | 30.11.2020 | <https://archive.vn/QEDPE> | 373,664 | 12,053 |
| 6 | [Canada](https://www2.deloitte.com/ca/en/pages/about-deloitte/articles/covid-dashboard.html) | National^a^ | Other | Deloitte. | Business decision-making, international organizations or those that depend on export markets | 17.07.2020 | <http://archive.vn/qrJbw> | NA | NA |
|  |  |  |  |  |  | 01.12.2020 | <https://archive.vn/E5b56> | 364,810 | 11,976 |
| 7 | [Newfoundland and Labrador](https://covid-19-newfoundland-and-labrador-gnl.hub.arcgis.com/) | Regional | Government | Government of Newfoundland and Labrador | General public of Newfoundland and Labrador^b^ | 20.07.2020 | <http://archive.vn/xv4sw> | 262 | 3 |
|  |  |  |  |  |  | 30.11.2020 | <https://archive.vn/YXOUf> | 337 | 4 |
| 8 | [Prince Edward Island](https://www.princeedwardisland.ca/en/information/health-and-wellness/pei-covid-19-case-data) | Regional | Government | Government of Prince Edward Island | General public of Prince Edward Island^b^ | 13.07.2020 | <http://archive.vn/oXtDj> | 33 | NA |
|  |  |  |  |  |  | 23.11.2020 | <https://archive.vn/YIC4d> | 68 | 3 |
| 9 | [Nova Scotia](https://novascotia.ca/coronavirus/data/) | Regional | Government | Government of Nova Scotia | General public of Nova Scotia^b^ | 16.07.2020 | <http://archive.vn/LOfCa> | 1,067 | 63 |
|  |  |  |  |  |  | 30.11.2020 | <https://archive.vn/kTtBU> | 1,257 | 65 |
| 10 | [New Brunswick](https://www2.gnb.ca/content/gnb/en/corporate/promo/covid-19.html) | Regional | Government | Government of New Brunswick | General public of New Brunswick^b^ | 20.07.2020 | <http://archive.vn/xEOZl> | 164 | 2 |
|  |  |  |  |  |  | 30.11.2020 | <https://archive.vn/bvwvF> | 495 | 7 |
| 11 | [Quebec](https://www.quebec.ca/sante/problemes-de-sante/a-z/coronavirus-2019/situation-coronavirus-quebec/) | Regional | Government | Government of Quebec | General public of Quebec | 14.07.2020 | <http://archive.vn/jky19> | 55,937 | 5,577 |
|  |  |  |  |  |  | 29.11.2020 | <https://archive.vn/Guu40> | 136,643 | 7,021 |
| 12 | [Quebec](https://www.inspq.qc.ca/covid-19/donnees) | Regional | Public health authority | National Institute of Public Health of Quebec | General public of Quebec^b^ | 14.07.2020 | [http://archive.vn/D3JfB on 14.07.20](http://archive.vn/D3JfB%20on%2014.07.20) | 56,730 | 5,633 |
|  |  |  |  |  |  | 30.11.2020 | <https://archive.vn/i3FwI> | 141,038 | 7,033 |
| 13 | [Montreal](https://santemontreal.qc.ca/en/public/coronavirus-covid-19/situation-of-the-coronavirus-covid-19-in-montreal/#c43674) | Municipal | Public health authority | Santé Montréal | General public of Montreal^b^ | 18.07.2020 | <https://archive.vn/Dfwbz> | 27,863 | 3,431 |
|  |  |  |  |  |  | 02.12.2020 | <http://archive.vn/KfneS> | 51,462 | 3,628 |
| 14 | [Ontario](https://covid-19.ontario.ca/data) | Regional | Government | Government of Ontario | General public of Ontario^b^ | 09.07.2020 | <http://archive.vn/1Urge> | 36,178 | 2,700 |
|  |  |  |  |  |  | 26.11.2020 | <https://archive.vn/htWsi> | 107,883 | 3,554 |
| 15 | [Ontario](https://www.publichealthontario.ca/en/data-and-analysis/infectious-disease/covid-19-data-surveillance/covid-19-data-tool) | Regional | Public health authority | Ontario Agency for Health Protection and Promotion | General public of Ontario^b^ | 16.07.2020 | <http://archive.vn/YtJVv> | 37,052 | NA |
|  |  |  |  |  |  | 30.11.2020 | <https://archive.vn/QK7RK> | 116,492 | 3,656 |
| 16 | [Ontario](https://howsmyflattening.ca/#/home) | Regional | Other | #HowsMyFlattening | Ontarians and public health leaders | 14.07.2020 | <http://archive.vn/7T6MT> | 36,950 | 2,722 |
|  |  |  |  |  |  | 24.11.2020 | <https://archive.vn/J5oAF> | NA | NA |
| 17 | [Toronto](https://www.toronto.ca/home/covid-19/covid-19-latest-city-of-toronto-news/covid-19-status-of-cases-in-toronto/) | Municipal | Public health authority | City of Toronto | Toronto Public Health and general public | 14.07.2020 | <http://archive.vn/NoyJh> | 14,735 | 1,110 |
|  |  |  |  |  |  | 23.11.2020 | <https://archive.vn/a4lFD> | 37,824 | 1,538 |
| 18 | [Ottawa](https://www.ottawapublichealth.ca/en/reports-research-and-statistics/daily-covid19-dashboard.aspx) | Municipal | Public health authority | Ottawa Public Health | General public of Ottawa^b^ | 16.07.2020 | <http://archive.vn/3ZL3k> | 2,167 | 263 |
|  |  |  |  |  |  | 30.11.2020 | <https://archive.vn/JGtyq> | 8,458 | 374 |
| 19 | [Manitoba](https://experience.arcgis.com/experience/f55693e56018406ebbd08b3492e99771) | Regional | Government | Government of Manitoba | General public of Manitoba^b^ | 18.07.2020 | <https://archive.vn/lT4SS> | 273 | 6 |
|  |  |  |  |  |  | 30.11.2020 | <https://archive.vn/XFXLo> | 16,483 | 301 |
| 20 | [Saskatchewan](https://dashboard.saskatchewan.ca/health-wellness) | Regional | Government | Government of Saskatchewan | Citizens of Saskatchewan | 20.07.2020 | <http://archive.vn/kVUii> | 943 | 15 |
|  |  |  |  |  |  | 30.11.2020 | <https://archive.vn/6najD> | 8,239 | 45 |
| 21 | [Alberta](https://www.alberta.ca/covid-19-alberta-data.aspx) | Regional | Government | Government of Alberta | Albertans | 18.07.2020 | <https://archive.vn/hQ9Kl> | 9,114 | 165 |
|  |  |  |  |  |  | 30.11.2020 | <https://archive.vn/AMWsk> | 56,444 | 533 |
| 22 | [British Columbia](https://experience.arcgis.com/experience/a6f23959a8b14bfa989e3cda29297ded) | Regional | Public health authority | Provincial Health Services Authority, BC Center for Disease Control | General public of British Columbia^b^ | 18.07.2020 | <http://archive.vn/Tspb3> | 3,198 | 189 |
|  |  |  |  |  |  | 30.12.2020 | <https://archive.vn/Vx7Hy> | 10,884 | 395 |
| 23 | [Vancouver](https://covid19dashboard.vancouver.ca/) | Municipal | Government | City of Vancouver | General public of Vancouver^b^ | 20.07.2020 | <http://archive.vn/jPIlp> | NA | NA |
|  |  |  |  |  |  | 30.11.2020 | <https://archive.vn/t3Eve> | NA | NA |
| 24 | [Yukon](https://yukon.ca/en/case-counts-covid-19) | Regional | Government | Government of Yukon | General public of Yukon^b^ | 13.07.2020 | <http://archive.vn/cG5a0> | 15 | NA |
|  |  |  |  |  |  | 23.11.2020 | <https://archive.vn/xKf7F> | 32 | 11 |
| 25 | [Northwest Territories](https://www.gov.nt.ca/covid-19/) | Regional | Government | Government of Northwest Territories | General public of Northwest Territories^b^ | 07.07.2020 | <http://archive.vn/UNPBb> | 5 | NA |
|  |  |  |  |  |  | 23.11.2020 | <https://archive.vn/74wC9> | 15 | 0 |
| 26 | [Nunavut](https://www.gov.nu.ca/health/information/covid-19-novel-coronavirus) | Regional | Government | Nunavut Department of Health | General public of Nunavut^b^ | 07.07.2020 | <http://archive.vn/HLobA> | 0 | 0 |
|  |  |  |  |  |  | 23.11.2020 | <https://archive.vn/iz5Bw> | 134 | 0 |

Notes: ^a^This dashboard is available for countries other than Canada. ^b^The intended audience was not explicitly stated and as such, the noted audience is inferred.

NA: not available.
