# Supplementary material for: Exploring Changes to the Actionability of COVID-19 Dashboards Over the Course of 2020 in the Canadian Context: Descriptive Assessment and Expert Appraisal Study
Source: J Med Internet Res. 2021 Aug 6;23(8):e30200. doi: 10.2196/30200 (PMC8360335; doi:10.2196/30200)
Supplement: Multimedia Appendix 3 [file jmir_v23i8e30200_app3.docx]

**Multimedia Appendix 3**

Scoring distribution and extent of agreement prior to joint workshops

| **Scorers** | **Distribution of dashboards** | | **Pre-panel scoring** | | **Proportion of scenarios on which the panel** | |
| --- | --- | --- | --- | --- | --- | --- |
|  | Dashboards discussed | Data points per reviewer | Partial/full agreements | Full agreements | Partially or fully agreed | Fully  agreed |
| NL, EB and DI | 1 | 14 | 10 | 8 | 71.43% | 57.14% |
| VB, EB and DI | 1 | 14 | 13 | 9 | 92.86% | 64.29% |
| MP, EB and DI | 5^a^ | 70 | 62 | 40 | 88.57% | 57.14% |
| KJG, EB and DI | 5^a^ | 70 | 60 | 35 | 85.71% | 50.00% |
| CW, EB and DI | 5^a^ | 70 | 62 | 36 | 88.57% | 51.43% |
| SW, EB and DI | 9 | 126 | 97 | 54 | 76.98% | 42.86% |
| **Total** | **26** | **364** | **304** | **182** | **83.52%** | **50.00%** |

^a^Dashboards discussed included a subset of dashboards originally scored by EB or DI.
